# Predictors of Remission or Combined Remission and Low Disease Activity in Rheumatoid Arthritis Patients in Taiwan: A Prospective Cohort Study

**DOI:** 10.3390/jcm13092521

**Published:** 2024-04-25

**Authors:** Ping-Han Tsai, Yao-Fan Fang, Yen-Fu Chen, Chih-Chieh Chen, Wen-Yu Chiang, Che-Tzu Chang, Yun-Ju Huang, Lieh-Bang Liou

**Affiliations:** 1 Division of Rheumatology, Allergy, and Immunology, Department of Internal Medicine, New Taipei Municipal Tucheng Hospital, New Taipei City 236, Taiwan; s001033@gmail.com (P.-H.T.); pisces4018@cgmh.org.tw (C.-C.C.); lugia7788@cgmh.org.tw (W.-Y.C.); 2 Division of Rheumatology, Allergy, and Immunology, Department of Internal Medicine, Chang Gung Memorial Hospital at Linkou, Taoyuan City 333, Taiwan; fang8802012@gmail.com (Y.-F.F.); patrichen0693@gmail.com (Y.-F.C.); chang3109@cgmh.org.tw (C.-T.C.); b9502071@cgmh.org.tw (Y.-J.H.); 3 School of Medicine, College of Medicine, Chang Gung University, Taoyuan City 333, Taiwan

**Keywords:** low disease activity, predictors, rheumatoid arthritis, remission

## Abstract

**Objectives**: This study aimed to identify predictors of remission or low disease activity (LDA) in patients with rheumatoid arthritis (RA) and low-ultrasound inflammation. **Methods**: A total of 80 patients with RA who fulfilled the 1987 ACR criteria for RA with a disease activity score of 28 joints (DAS28) > 3.2 were recruited. Over 1 year of therapy, we conducted blood tests every 6 months to examine erythrocyte sedimentation rate (ESR), C-reactive protein (CRP), monocyte chemotactic protein-1 (MCP-1), neuraminidase 3 (Neu3), and α-2,3-sialyltrasnferse I (ST3Gal-1) levels in B cells and monocytes. Additionally, we evaluated physical function by using the Health Assessment Questionnaire–Disability Index (HAQ-DI). Data on demographic and clinical parameters were collected, and musculoskeletal ultrasonography was performed twice a year on 12 specific joints to assess synovial changes. One year later, we compared all collected data and laboratory or ultrasound results between patients achieving remission or LDA and those who did not in order to determine the predictors. **Results**: Age, the presence or absence of rheumatoid factor, and the number of conventional disease-modifying anti-rheumatic drugs used were not correlated with remission or LDA for DAS28 or Simplified Disease Activity Index formulas. However, male sex, low CRP levels, low ESR levels, and low HAQ-DI scores were associated with a higher likelihood of achieving remission or LDA for DAS28-ESR. Negative anticyclic citrullinated peptide (CCP) and low HAQ-DI scores were predictors of remission or LDA for DAS28-MCP-1. Interestingly, having less than two comorbidities is a good predictor of a combined remission/low disease activity state for SDAI and DAS28-MCP-1. Furthermore, Neu3 and ST3Gal-1 levels and ST3Gal-1/Neu3 ratios in B cells and monocytes had no significant correlation with total ultrasound scores. Nevertheless, monocyte ST3Gal-1 and Neu3 correlated significantly with DAS28-ESR >5.1 and DAS-MCP-1 >4.8 (both categories belong to high disease activity), respectively (rho = 0.609 with *p* = 0.012, and rho = 0.727 with *p* = 0.011, respectively). Monocyte ST3Gal-1/Neu3 ratios connected with DAS28-ESR >5.1 and 3.3 < SDAI ≦ 11 (low disease activity), respectively (rho = 0.662 with *p* = 0.005, and rho = 0.342 with *p* = 0.048, respectively). **Conclusions**: In patients with RA in Taiwan, male sex, low CRP levels, low ESR levels, and low HAQ-DI scores are predictors of remission or LDA for DAS28-ESR, which differ from the predictors for DAS28-MCP-1. Moreover, monocyte ST3Gal-1, Neu3, and their ratios correlated with different disease activity categories of DAS28-ESR, DAS28-MCP-1, and SDAI scores.

## 1. Introduction

Rheumatoid arthritis (RA) is a systemic disease primarily affecting limb joints, resulting in the loss of normal joint function and decreased daily activity. Ultimately, it can lead to a decline in overall life function and occupational capability [1,2]. Approximately 1% of the Taiwanese population is affected by RA [3]. Continuous high disease activity is associated with progressive joint destruction, whereas low disease activity (LDA) or remission indicates low radiographic damage [4,5,6]. Therefore, achieving remission or LDA is considered an ideal goal for favorable outcomes [5]. Because the disease activity score using the 28-joint erythrocyte sedimentation rate (DAS28-ESR) remission score, defined as <2.6, may not indicate disease remission correctly [7], subsequent modifications and alternative methods have been developed [8,9,10,11].

Magnetic resonance imaging (MRI) examinations have revealed the silent progression of bone erosion in 40% of patients with RA with improved DAS28-ESR and DAS28-C-reactive protein (CRP) scores [12]. Achieving the lowest disease activity, particularly remission, is considered highly desirable. However, establishing standardized MRI remission cutoff values remains a challenge [12]. Moreover, MRI may not be cost-effective. Exploring the potential value of using another laboratory biomarker, such as monocyte chemotactic protein-1 (MCP-1), to replace ESR in the original DAS28 formula for defining remission is a subject worth further examination [11]. MCP-1 is a crucial chemokine responsible for regulating the migration and infiltration of monocytes/macrophages [13]. It is implicated in the pathogenesis of fibrosis in systemic sclerosis [14], and is associated with psoriasis [15] and rheumatoid arthritis [16]. MCP-1 is locally produced at the site of inflammation by activated monocytes and fibroblasts [17], potentially providing a more accurate differentiation between remission and non-remission. In our previous study [11], we reported a high correlation between DAS28-MCP-1 scores and DAS28-ESR and DAS28-CRP scores, with correlation coefficients of 0.984 and 0.971, respectively. Notably, our research indicated that DAS28-MCP-1 exhibited a significantly higher remission rate than DAS28-ESR, according to two different remission definitions. Furthermore, it demonstrated a significantly higher remission rate than the Simplified Disease Activity Index (SDAI) based on the 2005 modified American Rheumatism Association remission criteria [18,19,20]. Identifying predictors of remission in RA is crucial for guiding treatment decisions in patients with RA. Although various factors associated with remission have been reported in the literature, a consensus on the most effective predictor is lacking. Furthermore, findings on these predictors have not been reported in Taiwanese studies. Moreover, predictors of remission have not been assessed against DAS28-MCP-1-based remission.

α-2,3-sialyltransferase I (ST3Gal-1) catalyze α-2,3 sialylation to Gal β1,3 GalNAc, which is engaged in T cell apoptosis and tumorigenesis [21,22,23,24]. ST3Gal-1 is likely confined in the Golgi apparatus and cell membrane. In contrast, neuraminidases desialylate sialic acid in cells. Neuraminidase-3 (Neu3), the ganglioside-specific sialidase, may play a crucial role in cell-surface events by modulating gangliosides [25]. Neu3 is exclusively found on the cell surface [25]. There is currently a gap in knowledge regarding whether the levels of Neu3 and ST3Gal-1 in B cells and monocytes can predict RA remission. This study aims to investigate whether the levels of these enzymes can predict RA remission, as their levels in monocytes are correlated with DAS28-ESR scores >5.1 [26]. Specifically, multivariable analysis revealed that monocyte ST3Gal-1 levels are significantly associated with DAS28-ESR scores but not with CRP and ESR in RA patients with DAS28-ESR > 5.1, especially not for those with ≤5.1 [26]. Based on these findings, we decided to assess monocyte ST3Gal-1 and Neu3 levels as potential predictors of RA remission. Interestingly, B cell ST3Gal-1 and Neu3 levels also correlated with DAS28-ESR scores >5.1, but not with those ≤5.1 [27]. Therefore, in this study, we also evaluated B cell ST3Gal-1 and Neu3 levels as predictor candidates. Also, studies have suggested that the concurrent reverse expression of α2,6-sialic acid (SIA) ratios on IgM and IgG is correlated with the occurrence of collagen-induced arthritis and RA disease activity [27]. Therefore, α2,6-SIA ratios on IgG-anticyclic citrullinated peptide (CCP) antibodies and IgM-rheumatoid factor (RF) are potential markers for evaluating RA disease activities [27]. Furthermore, whether predictors of remission against DAS28 or SDAI-based remission closely align with predictors of remission against ultrasound-detected none or minimal synovitis remains unknown.

The primary objective of this study was to identify predictors of RA remission. Additionally, the study examined variations in predictors of RA remission based on different disease activity measures and validated these predictors through ultrasound examination, specifically assessing anatomic none/minimal synovitis. This comprehensive approach provides rheumatologists with improved clinical predictors of remission, enhancing their ability to effectively predict treatment responses.

## 2. Methods

### 2.1. Study Design, Data Sources, Study Population, and Case Definition

#### 2.1.1. Participants and Study Design

The study was conducted at New Taipei Municipal Tucheng Hospital and the Lin-kou and Taipei branches of Chang Gung Memorial Hospital, Taiwan, spanning from January 2021 to October 2023. The study protocol was approved by the Institutional Review Board of Chang Gung Memorial Hospital. A total of 80 patients with RA who met the 1987 ACR criteria for RA with DAS28 > 3.2 (indicating moderate and high disease activity) were recruited as candidates after they provided written informed consent. The projected number of 80 patients with RA was determined on the basis of the number of patients from our most recent study results. Patients with RA between 20 and 80 years were randomly enrolled, including both consecutive and volunteered participants from our rheumatology outpatient departments. Follow-up assessments, conducted every 6 months over a 12-month period (3 visits per patient), involved various evaluations: blood collection, clinical assessment, and ultrasound examination (performed twice annually). A blood hs-CRP level <5 mg/L and an ESR < 20 mm/h for women aged ≤50 years and <30 mm/h for women aged >50 years were considered normal levels, as determined by our hospital laboratory, and these parameters were monitored at each visit.

The primary objective of this study was to investigate predictors of remission in RA in the Taiwanese population. We collected comprehensive data on various factors to elucidate conditions predicting remission in RA. These factors included chemokine status (MCP-1), cell-surface molecules (ST3Gal-1, Neu3), clinical parameters (age, sex, body mass index [BMI], smoking status, disease activity and disability, and conventional disease-modifying anti-rheumatic drugs [cDMARDs]), and levels of ESR, CRP, RF, and anti-CCP. Notably, the analysis does not incorporate the utilization of biologic disease-modifying anti-rheumatic drugs due to their consistent administration to all patients. Our secondary objective was to compare the differences in predictors of remission by using various clinical evaluation tools (DAS28-ESR, DAS28-MCP-1, DAS28-CRP, and SDAI). Additionally, we aimed to validate predictors of remission against ultrasound results. A total of 8 mL of blood was obtained from these patients with RA and tested for chemokines and molecules. The objectives include the following: (1) to examine MCP-1 levels and ST3Gal-1 and Neu3 from B cells and monocytes to clarify their roles as predictors of remission in patients with RA and (2) to compare predictors of remission defined by the remission criteria of different disease activity measures (DAS28-ESR, DAS28-MCP-1, DAS28-CRP, and SDAI). Medications were administered at the discretion of individual rheumatologists.

#### 2.1.2. Cell Separation and Plasma Collection

Blood samples were collected in tubes containing ethylenediaminetetraacetic acid and centrifuged, after which the cells were collected. Further separation procedures were employed to isolate peripheral blood mononuclear cells (PBMCs) [28].

#### 2.1.3. Examination of ST3Gal-1 and Neu3 on Monocytes and B Lymphocytes

PBMCs were prepared in a solution of 100 μL of phosphate-buffered saline (PBS) with 1% bovine serum albumin (Sigma, St. Louis, MO, USA). The cells were then stained with phycoerythrin (PE)-mouse anti-human CD14 (clone: M5E2) for monocytes and PE-mouse anti-human CD19 (clone: HIB19) for B cells (used at manufacturer’s suggested dilutions) (BD Pharmingen, Mountain View, CA, USA). The staining process occurred in low-light conditions for 1 h at room temperature (20–25 °C). For the isotype control, PE-mouse IgG1 *κ* isotype control (clone: MOPC-21) was used for anti-CD14 and anti-CD19 (used at the manufacturer’s suggested dilutions; BD Pharmingen, CA, USA). Additionally, rabbit polyclonal IgG anti-ST3Gal-1 (0.6 μg/mL) and rabbit polyclonal IgG anti-Neu3 (0.4 μg/mL; Abcam, Cambridge, MA, USA) were used to stain the cells. Rabbit polyclonal IgG (Jacskon ImmunoResearch, West Grove, PA, USA) (2 μg/mL; Sigma, St. Louis, MO, USA) served as the control.

Following washing and centrifugation at 644× *g* using the Eppendorf centrifuge 5810R (Hamburg, Germany) for 5 min, cells suspended in 100 μL of PBS were subsequently stained with allophycocyanin (APC)–goat polyclonal antibody and rabbit IgG (2 μg/mL; Abcam, Cambridge, MA, USA). This staining process was conducted in low-light conditions at room temperature (20–25 °C) for 1 h. After staining, using similar washing and centrifugation techniques, the cells were resuspended in 500 μL of PBS at 4 °C for flow cytometric analysis using the BD FACSCalibur System. A minimum of 20,000 cells were collected, and the FlowJo 7.6.1 program (FlowJo, LLC, Ashland, OR, USA) was used in the analysis of flow cytometry data.

#### 2.1.4. Plasma Examination of MCP-1 in Patients with RA

The plasma from the enrolled patients with RA was analyzed for MCP-1, following the described protocol [20].

#### 2.1.5. Clinical Assessments in 80 Patients with RA

During each visit, clinicians collected data on the patients’ current medications, Health Assessment Questionnaire–Disability Index (HAQ-DI) items [29,30], morning stiffness, tender joint count (TJC), swollen joint count (SJC), patients’ and evaluators’ global assessment of disease activity (measured using a visual-analog scale [VAS; in cm] as the patient’s global assessment [PGA] and evaluator’s global assessment [EGA]), ESR, and CRP.

#### 2.1.6. Measurement of Serum Laboratory Parameters in 80 Patients with RA

During the first visit (baseline), RF levels were measured through nephelometry by using the N Latex RF Kit from Siemens Healthcare Diagnostics Products, Marburg, Germany). Additionally, anti-CCP antibodies (Quanta Lite CCP3 IgG ELISA kit; Inova Diagnostics, Inc., San Diego, CA, USA) were examined twice in 12 months (at baseline and month 12).

#### 2.1.7. Calculation of Different Disease Activity Score28 (DAS28) Definitions

The DAS28-ESR score, DAS28-CRP score, and SDAI, were calculated using the following formulas, as previously described [31]: DAS28-ESR = [0.56 × √TJC] + [0.28 × √SJC] + 0.70 × ln[ESR] + 0.014 × PGA [in mm]; DAS28-CRP = [0.56 ×√TJC] + [0.28 ×√SJC] + (0.36 × ln [CRP; in mg/L]) + 1) + (0.014 × PGA [in mm]) + 0.96); and SDAI = SJC + TJC + PGA [VAS; in cm] + EGA [VAS; in cm] + CRP [in mg/dL]. DAS28-MCP-1 scores were calculated using a modified formula: DAS28-MCP-1 = 0.56 × √TJC + 0.28 × √SJC + 0.39 × ln(MCP-1) + 0.014 × (PGA [in mm]) [11]. Remission endpoints were defined as DAS28 < 2.6, DAS28-CRP < 2.4, DAS28-MCP-1 < 2.2, and SDAI ≤ 3.3. Moreover, LDA was designated as: 2.6 ≤ DAS28-ESR ≤ 3.2, 2.4 ≤ DAS28-CRP ≤ 2.9, 2.2 ≤ DAS28-MCP-1 ≤ 3.6, and 3.3 < SDAI ≤ 11. Moderate disease activity for RA was defined as 3.6 < DAS28-MCP-1 ≤ 4.8, and high disease activity was indicated by DAS28-MCP-1 > 4.8.

#### 2.1.8. Ultrasound Examination of 80 Patients with RA

The ultrasound measurements were conducted by three rheumatologists who had received comprehensive training in musculoskeletal ultrasound techniques. To maintain consistency and reliability in the measurements, the rheumatologists rotated in performing assessments. Before initiating the study, a consensus meeting was held among the rheumatologists to establish standardized protocols for the scoring assessments. The musculoskeletal ultrasound machine used in our study was the Siemens Acuson P300 Portable Ultrasound Machine(Siemens Medical Solutions USA, Inc., CA, USA). The ultrasound score system employed in this study is based on the EULAR-OMERACT scoring system, where grade 0 indicates a normal joint, grade 1 denotes minimal synovitis, grade 2 indicates moderate synovitis, and grade 3 denotes severe synovitis [32]. The evaluation of none or minimal synovitis (grade 0/grade 1) was employed to validate predictors of remission obtained earlier against DAS28 score remission definitions. Patients underwent examinations for both grayscale and power Doppler components, as well as their combination, at the wrist, 2/3 MCP, elbow, knee, and ankle joints at baseline and month 12. Total ultrasound scores were defined by us as the sum of grayscale and power Doppler of all examined joints. This approach is innovative; specifically, the extent of ultrasound-defined synovitis has not been utilized to validate predictors of remission defined with respect to different disease activity measures.

### 2.2. Statistical Analysis

The odds ratio was used to compare baseline parameters between DAS28 remission and combined remission/low disease activity (LDA) endpoints. The latter combination denotes scores of DAS28-ESR ≤ 3.2, DAS28-CRP ≤ 2.9, DAS28-MCP-1 ≤ 3.6, and SDAI ≤ 11. Each baseline predictor was treated as either continuous (e.g., BMI, RF, anti-CCP, CRP, ESR, MCP-1, DAS28, and HAQ-DI) or categorical (e.g., age > 40, vs. ≤40, >40 to ≤65 vs. ≤40, ≥65 vs. <65 years), anti-CCP and RF status, and positive vs. negative) as described previously [33]. All statistical calculations are performed based on each individual visit, rather than on different patients. *p* < 0.05 indicated statistical significance.

## 3. Results

Table 1 presents the summary statistics of the demographic characteristics, laboratory findings, and the number of RA patients using conventional disease-modifying anti-rheumatic drugs (cDMARDs), biologic disease-modifying antirheumatic drugs (bDMARDs), and targeted disease-modifying antirheumatic drugs (tDMARDs), as well as the dosage. Among the patients with RA, 69 were female and 11 were male, with an average age of 56.0 ± 10.6 years and a disease duration of 115.38 ± 77.98 months. The baseline TJC was 4.1 ± 3.5, and SJC was 3.4 ± 2.8. The HAQ-DI score was 0.7 ± 0.9, and the BMI was 24.7 ± 4.3. Among the comorbidities, 21 patients had hypertension (26.26%), 3 patients had type 2 diabetes mellitus (3.75%), 6 patients had chronic kidney disease (7.5%), and 1 patient had a cerebrovascular accident (1.25%). From a total of 80 patients, data from 182 visits were ultimately obtained. However, for some patients, data for the 6th or 12th month was not collected due to the patients’ noncompliance with blood tests.

Table 2 presents a comparison of various physiological, biochemical, drug-related, and comorbidity factors related to disease remission defined on the basis of different DAS28 score–based remission criteria (see criteria in Methods). Among all the factors considered, sex, age, the presence or absence of autoantibodies (RF, anti-CCP), the level of inflammation markers (ESR, CRP), HAQ-DI scores, the use of cDMARDs, ultrasound grade, use of cDMARD, bDMARDs, and tDMARDs, and comorbidities exhibited no difference between cutoff categories for remission in disease activity. Nevertheless, normal BMI was the only predictor for achieving DAS28-CRP remission <2.4, SDAI remission ≤3.3, and DAS28-MCP-1 remission <2.2 (Table 2).

Table 3 presents a comparison of various physiological, biochemical, drug-related, and comorbidity factors related to remission or LDA across different DAS28 scores (definition for combined remission and LDA: DAS28-ESR ≤ 3.2, DAS28-CRP ≤ 2.9, DAS28-MCP-1 ≤ 3.6, and SDAI ≤ 11). Among all the factors analyzed, male sex, negative anti-CCP, low CRP and ESR levels, and a low HAQ-DI level were identified as being more likely to achieve combined remission and LDA. Specifically, male sex, low CRP, low ESR, and a low HAQ-DI score were predictors of combined remission and LDA for DAS28-ESR. Negative anti-CCP and low HAQ-DI scores were predictors of combined remission and LDA for DAS28-MCP-1. Notably, only a low HAQ-DI score was a predictor of combined remission and LDA for SDAI (Table 3). Interestingly, having less than two comorbidities is a good predictor of combined remission/low disease activity state for SDAI and DAS28-MCP-1.

**Figure 1:**
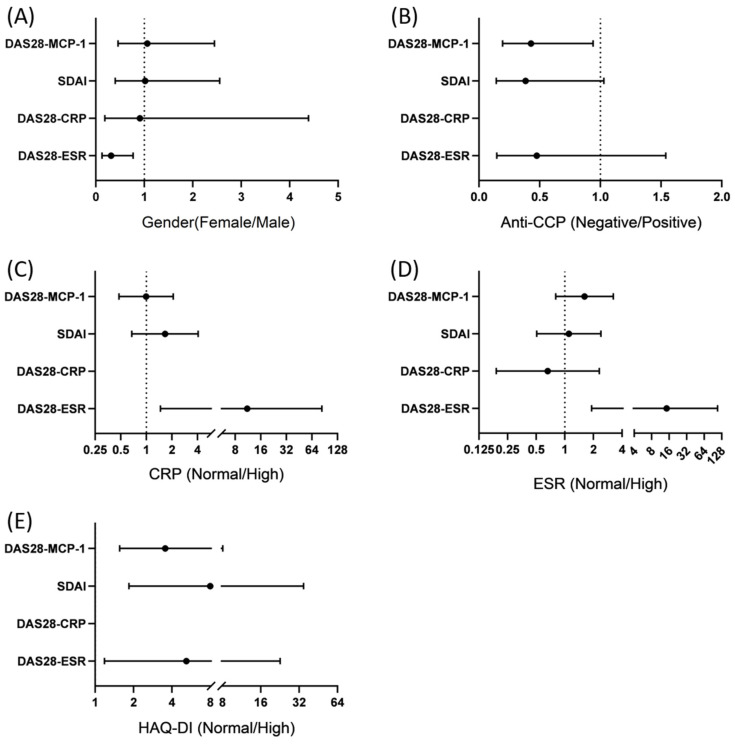
Predictors for combined remission and low disease activity (LDA) among different DAS28 scores. Comparison of (**A**) sex (female vs. male); *p* = 0.009 for remission/LDA of DAS28-ESR. (**B**) Anti-CCP (negative/positive); *p* = 0.033 for remission/LDA of DAS28-MCP-1. (**C**) CRP (normal/high); *p* = 0.002 for remission/LDA of DAS28-ESR. (**D**) ESR (normal/high); *p* < 0.001 for remission/LDA of DAS28-ESR. (**E**) HAQ-DI (normal/high); *p* = 0.002, 0.001, and 0.018 for remission/LDA of DAS28-MCP-1, of SDAI, and of DAS28-ESR, respectively.

To enhance the clarity of the information provided in Table 3, a visual representation is presented in Figure 1. Figure 1A presents the female to male odds ratios, which were significant only for DAS28-ESR < 3.2, with *p* values >0.05 for the other three categories. Figure 1B presents the odds ratios of anti-CCP (negative to positive), indicating significance only for DAS28-MCP-1 ≤ 3.6 (No DAS28-CRP line is included in Figure 1B, as no patient fulfilled the criteria for anti-CCP discrimination in combined remission and LDA, thereby preventing the calculation of odds ratios.). Figure 1C presents the odds ratios of normal CRP to high CRP, indicating significance only for DAS28-ESR ≤ 3.2. Figure 1D presents the odds ratios of normal ESR to high ESR; only DAS28-ESR ≤ 3.2 was significant. Figure 1E presents the odds ratios of normal HAQ-DI to high HAQ-DI, with significance only for DAS28-MCP-1 ≤ 3.6, SDAI ≤ 11, and DAS28-ESR ≤ 3.2 (no DAS28-CRP line is included in Figure 1E, as no patient fulfilled the criteria for HAQ-DI discrimination in remission or LDA, precluding the calculation of odds ratios).

Table 4 (A) presents the correlation between ST3Gal-1 and Neu3 levels in B cells and monocytes with the total ultrasound score. The analysis revealed that neither the levels of Neu3 and ST3Gal-1 in B cells and monocytes nor the ratio of ST3Gal-1/Neu3 were significantly correlated with the total ultrasound score. Table 4 (B) presents a comparison of ST3Gal-1 and Neu3 in B cells and monocytes at different ultrasound grades (low grade 0/1 vs. high grade 2/3). Overall, no significant difference was noted in the surface enzymes of B cells or monocytes between the severity of ultrasound findings (low grade 0/1 vs. high grade 2/3).

**Figure 2:**
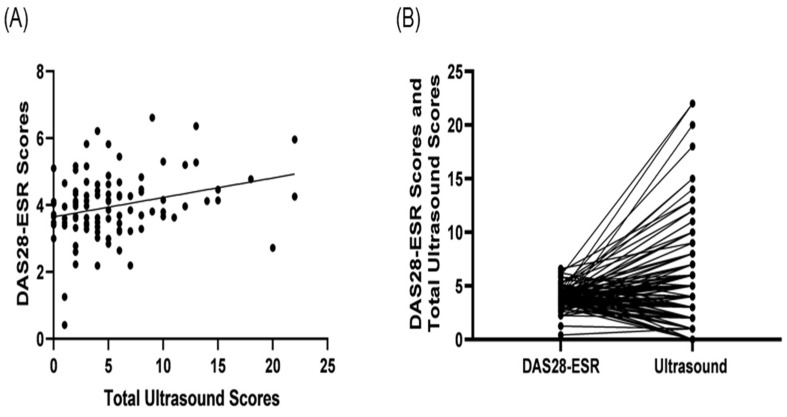
Correlation between total ultrasound scores and DAS28-ESR scores. (**A**) Total ultrasound scores: the sum of combined grayscale and power Doppler score. The Mann–Whiney U test (*n* = 104) gave r = 0.262 and *p* = 0.008. (**B**) DAS28-ESR scores were displayed to pair with the total ultrasound scores (*n* = 104) at t = 3.055 and *p* = 0.003.

The study also conducted a correlation analysis between the total ultrasound score and the different RA disease activity scores (DAS28-ESR, DAS28-CRP, SDAI, and DAS28-MCP-1; Table 5). Figure 2A,B demonstrated the results of the total ultrasound Scores and its correlation and pairness with DAS28-ESR scores. The results indicated a significant correlation between the total ultrasound scores and DAS28-ESR, but not for other formula scores.

Nevertheless, among different disease activity categories, monocyte ST3Gal1 levels and monocyte ST3Gal1/Neu3 ratios exhibited a positive correlation with DAS28-ESR > 5.1 (high disease activity; Table 6), consistent with our previously reported results [16]. Similarly, monocyte ST3Gal1/Neu3 ratios demonstrated a positive correlation with 3.3 < SDAI ≤ 11 (LDA). Moreover, monocyte Neu3 levels exhibited a positive correlation with DAS28-MCP-1 > 4.8 (high disease activity; Table 6).

Table 7 presents the clinical, laboratory, and ultrasound data at different stages of the disease progression. At month 0 (M0), month 6 (M6), and month 12 (M12), the numbers of patients (*n*) were 80, 52, and 50, respectively. The data includes CRP levels (mg/L), ESR (mm/h), RF (IU/mL), Anti-CCP (CU), TJC, SJC, DAS28-ESR, DAS28-CRP, SDAI, DAS28-MCP-1, HAQ-DI, and total ultrasound scores. Notably, at month 12, the ultrasound score data is based on 48 patients. Additionally, the table includes data on B cell ST3, B cell Neu3, monocyte ST3, monocyte Neu3, B cell ST3/Neu3 ratios, and monocyte ST3/Neu3 ratios, providing comprehensive insights into the disease progression at different time points. The distribution of B cell ST3 and Neu3, as well as monocyte Neu3, shows a negative trend associated with ESR, CRP, and various disease activity formula scores. However, monocyte ST3 decreases sequentially at M0, M6, and M12.

## 4. Discussion

Our study findings revealed that predictors of combined remission and LDA for DAS28-ESR (male sex, low CRP levels, low ESR levels, and low HAQ-DI scores) differ from those for DAS28-MCP-1 (negative anti-CCP and low HAQ-DI scores; Table 3). This distinction underscores the need to recognize the distinct factors influencing the achievement of combined remission and LDA for DAS28-ESR compared with DAS28-MCP-1. Additionally, BMI levels significantly differentiate DAS28-MCP-1 remission, DAS28-CRP remission, and SDAI remission from non-remission, in contrast to the scenario observed for DAS28-ESR (Table 2). This implies that BMI is not a predictor of remission for DAS28-ESR.

The levels of ST3Gal-1 and Neu3, as well as the ST3Gal-1/Neu3 ratios in B cells and monocytes and different DAS28 formula scores, exhibited no correlation with the total ultrasound score. Furthermore, no significant correlation was observed between the severity of ultrasound findings (low grade 0/1 vs. high grade 2/3) and the surface enzymes of B cells or monocytes. These findings, although preliminary, may offer valuable insights for physicians in the treatment of patients with RA, provided that a larger patient cohort can be enrolled.

Remission of RA has been reported to be associated with various factors, including younger age [33,34,35], body weight (positive association with low BMI [33] and negative association with obesity [35,36]), smoking history [35], male sex [34,35,37], elevated ESR at the time of diagnosis [35], disease activity status (lower tender joint score at baseline [38], lower DAS-28 score at baseline [34,37,39], lower HAQ score at baseline [33,37,39,40,41], and higher baseline EGA and PGA [27]), better functional status [35], autoantibodies status (ACPA positivity [39,42]) and biomarkers status (including serum MMP-3 [43,44], IL-17 [45], IL-6 [45], VEGF [45], TNF-α [45], 14-3-3η [46], reactive oxygen metabolites (ROMs) [41], and multi-biomarker disease activity score [47]), inflammation evidence on clinical image examinations (baseline MRI osteitis and tenosynovitis [48]), hematological parameters (Hb level, NLR, and MPV [49]), higher education level [37], early diagnosis of RA [50], early intervention with DMARD [51], alcohol consumption [52], and baseline RA medication prescription on disease activity severity [52].

Our study results align with previous findings, indicating that male sex [34,35,37], ACPA positivity [39,42], low ESR at the time of diagnosis [35], and lower HAQ score at baseline [33,37,39,40,41] are associated with achievement of combined remission and LDA after treatment. Moreover, our study demonstrated that low HAQ-DI scores predicted remission in DAS28-CRP, SDAI, and DAS28-MCP-1 (Table 2). Since MCP-1 plays a significant role in inflammation in RA [53], and prognostic factors may be correlated with DAS28-MCP-1 [54], our study further investigates factors associated with prognosis improvement in DAS28-MCP-1. Previous research in this area has been limited, and our study contributes to expanding knowledge in this field. However, some of our study findings diverge from those reported in the literature. For example, our study revealed that the number of cDMARDs used at baseline was not associated with remission or LDA after treatment, which contradicts the TACERA longitudinal cohort study [52] from the United Kingdom. The TACERA cohort, with a larger patient population (275 patients) and different from our study in terms of ethnicity (mostly white ethnicity in the TACERA trial and Han population in our study), primarily focused on comparing the prescription of methotrexate alongside a second DMARD against methotrexate monotherapy, excluding steroid use, and its association with achieving SDAI remission at 6 months. By contrast, our study mainly compared the use of less than two types with more than two types of cDMARDs. Additionally, our study revealed that the presence or absence of RF did not exhibit any correlation with remission or LDA in RA, a finding different from previous studies. Furthermore, given that the average duration of disease in our study is 9.5 years, our research findings reflect the corresponding relationships between various disease activity formula scores and cell surface biomarkers in patients with long-term chronic rheumatoid arthritis.

Our study findings indicate that the levels of ST3Gal-1, Neu3, and ST3Gal-1/Neu3 ratios in B cells and monocytes, along with various DAS28 formula scores, did not exhibit any correlation with the total ultrasound score and different ultrasound severity levels. This novel perspective, not reported previously, highlights the need for further patient follow-up to address the current research limitations in this field. In a previous study, ST3Gal-1 and Neu 3 of monocytes were correlated with DAS28-ESR as measures of RA disease activity [26]. Additionally, our study observed a tendency where the distribution of B cell ST3 and Neu3, as well as Monocyte Neu3, shows a negative trend associated with ESR, CRP, and various disease activity formula scores. However, the sequential decrease in monocyte ST3 at M0, M6, and M12 suggests that this may have the potential to become a biomarker for monitoring disease activity in rheumatoid arthritis.

Our study has several limitations that should be considered. First, the not-a-large sample size may hinder achieving statistical significance in the correlation of various remission factors. Nevertheless, similar studies in the past that have demonstrated clinical significance for predictors of remission with not-large sample sizes and their patient numbers (70, 75, and 74, respectively) are similar to the enrolled patient number in our study [45,46,47,48,49,50,51,52,53]. Second, the effects of smoking history and alcohol consumption were not analyzed in our study because of the small number of patients with smoking habits in our sample (5 out of 80 patients, with 3 smoking 1 pack per day and the other 2 smoking 2 packs per day) and alcohol consumption (2 out of 80 patients with regular alcohol consumption). Consequently, analyzing the correlation of smoking and alcohol consumption with remission or LDA in patients with RA becomes challenging due to the limited representation in the sample.

In conclusion, this prospective study in Taiwan is the first of its kind and reports that male sex, low CRP levels, low ESR levels, and low HAQ-DI scores are predictors of combined remission and LDA in DAS28-ESR. These predictors differ from those associated with combined remission and LDA (negative anti-CCP and low HAQ-DI scores) for DAS28-MCP-1. Moreover, low HAQ-DI scores predicted remission or LDA for DAS28-ESR, SDAI, and DAS28-MCP-1, but not for DAS28-CRP. Moreover, having less than two comorbidities in RA patients is a good predictor of combined remission/low disease activity state for SDAI and DAS28-MCP-1. Therefore, patients with RA exhibiting these characteristics are expected to experience improved treatment outcomes in Taiwan.

## Figures and Tables

**Table 1:** Demographic, laboratory data and medications of patients with RA at baseline (day 0).

	Mean ± S.D.	Range
Gender	F:M = 69:11	
Age	56.0 ± 10.6	30–74
Duration of RA (months)	115.38 ± 77.98	15–277
CRP (mg/L)	4.50 ± 10.36	0.2–88.86
ESR (mm/h)	25.7 ± 25.0	1–140
RF (IU/mL)	162.3 ± 269.7	5–1200
Anti-CCP (CU)	705.8 ± 1065.9	4.6–6017.8
TJC	4.1 ± 3.5	0–17
SJC	3.4 ± 2.8	0–11
HAQ-DI score	0.7 ± 0.9	0–4
BMI	24.7 ± 4.3	16.6–40.7
Corticosteroids		
Prednisolone (*n* = 27, 33.75%)	4.8 ± 1.8 (mg/day)	2.5–10
Methylprednisolone (*n* = 2, 2.5%)	3 ± 1.4 (mg/day)	2–4
cDMARDs		
Methotrexate (*n* = 57, 71.25%)	11.4 ± 3.4 (mg/week)	5–20
Sulfasalazine (*n* = 29, 36.25%)	2052.6 ± 868.3 (mg/day)	1000–3000
Hydroxychloroquine (*n* = 23, 28.75%)	287.0 ± 96.8 (mg/day)	200–400
Leflunomide (*n* = 14, 17.5%)	19.3 ± 2.7 (mg/day)	10–20
Azathioprine (*n* = 9, 11.25%)	138.9 ± 48.6 (mg/day)	100–200
Cyclosporine (*n* = 3, 3.75%)	133.3 ± 57.7 (mg/day)	100–200
bDMARDs		
Etanercept (*n* = 3, 7.25%)	50 (mg/week)	-
Adalimamab (*n* = 4, 5%)	70 ± 20 (mg/month)	40–80
Golimumab (*n* = 6 7.5%)	50 (mg/month)	-
Certolizumab (*n* = 19, 23.75%)	400 (mg/month)	-
Tocilizumab (*n* = 11, 13.75%)	610.9 ± 78.2 (mg/month)	400–648
Abatacept (*n* = 9, 11.25%)	527.8 ± 83.3 (mg/month)	500–750
tDMARDs		
Tofacitinib (*n* = 8, 10%)	11.6 ± 3.0 (mg/day)	10–19
Baricitinib (*n* = 8, 10%)	4 (mg/day)	-
Upadacitimib (*n* = 1, 1.25%)	15 (mg/day)	-
Comorbidity		
Hypertension (*n* = 21, 26.26%)		
Type 2 diabetes mellitus (*n* = 3, 3.75%)		
Chronic kidney disease (*n* = 6, 7.5%)		
Cerebrovascular accident (*n* = 1, 1.25%)		

CRP: C-reactive protein; ESR: erythrocyte sedimentation rate; RF: rheumatoid factor; anti-CCP: anticyclic citrullinated peptide. TJC: tender joint count; SJC: swollen joint count; HAQ-DI: Health Assessment Questionnaire–Disability Index. cDMARDs: conventional disease-modifying anti-rheumatic drugs; bDMARDs: biologic disease-modifying antirheumatic drugs; tDMARDs: targeted disease-modifying anti-rheumatic drugs. Normal ranges: CRP < 5 mg/L; ESR < 20 mm/h for women aged ≤50 years and <30 mm/h for women aged >50 years; ESR < 15 mm/h for men aged ≤50 years and <20 mm/h for men aged >50 years; RF < 15 IU/mL; anti-CCP ≤ 20 CU.

**Table 2:** Comparative analysis of physiological, biochemical, drug-related, and comorbidity factors in remission states based on different DAS28 scores.

	DAS28-ESR	DAS28-CRP	SDAI	DAS28-MCP-1
Female/Male	OR = 0.457 (*p* = NS)	Infinity (*p* = NS)	OR = 1.095 (*p* = NS)	OR = 1.607 (*p* = NS)
Age (<40/>41)	Infinity (*p* = NS)	Infinity (*p* = NS)	Infinity (*p* = NS)	Infinity (*p* = NS)
Age (<40/41–64)	Infinity (*p* = NS)	Infinity (*p* = NS)	Infinity (*p* = NS)	Infinity (*p* = NS)
Age (<64/>65)	OR = 2.761 (*p* = NS)	Infinity (*p* = NS)	Infinity (*p* = NS)	Infinity (*p* = NS)
RF (Negative/positive)	OR = 1.565 (*p* = NS)	OR = 0.511 (*p* = NS)	Infinity (*p* = NS)	OR = 0.238 (*p* = NS)
Anti-CCP (Negative/positive)	OR = 1.333 (*p* = NS)	Infinity (*p* = NS)	Infinity (*p* = NS)	Infinity (*p* = NS)
CRP (Normal/high)	Infinity (*p* = NS)	Infinity (*p* = NS)	Infinity (*p* = NS)	OR = 1.046 (*p* = NS)
ESR (Normal/high)	Infinity (*p* = NS)	OR = 0.321 (*p* = NS)	OR = 0.434 (*p* = NS)	OR = 0.480 (*p* = NS)
HAQ-DI (Normal/high)	Infinity (*p* = NS)	Infinity (*p* = NS)	Infinity (*p* = NS)	Infinity (*p* = NS)
Medication numbers (≦2/>2)	OR = 1.863 (*p* = NS)	OR = 2.637 (*p* = NS)	OR = 2.048 (*p* = NS)	OR = 1.578 (*p* = NS)
BMI (Normal/abnormal)	OR = 0.670 (*p* = NS)	Infinity (*p* = 0.008 **)	Infinity (*p* = 0.016 *)	Infinity (*p* = 0.002 **)
Ultrasound grade (≦1/>1)	OR = 2.717 (*p* = NS)	Infinity (*p* = NS)	OR = 2.717 (*p* = NS)	OR = 1.840 (*p* = NS)
Anti-TNF (User/non-user)	OR = 1.246 (*p* = NS)	OR = 2.627 (*p* = NS)	OR = 1.009 (*p* = NS)	OR = 1.015 (*p* = NS)
Anti-IL-6 (User/non-user)	OR = 2.460 (*p* = NS)	OR = 2.870 (*p* = NS)	OR = 2.891 (*p* = NS)	OR = 2.804 (*p* = NS)
JAKi (User/non-user)	OR = 0.367 (*p* = NS)	Infinity (*p* = NS)	OR = 0.685 (*p* = NS)	OR = 0.921 (*p* = NS)
CTLA4-Ig (User/non-user)	OR = 0.527 (*p* = NS)	Infinity (*p* = NS)	OR = 0.571 (*p* = NS)	Infinity (*p* = NS)
cDMARDs (MTX/non-MTX)	OR = 0845 (*p* = NS)	OR = 1 (*p* = NS)	OR = 1.165 (*p* = NS)	Infinity (*p* = NS)
Comorbidities (<2/≧2)	OR = 0.736 (*p* = NS)	Infinity (*p* = NS)	Infinity (*p* = NS)	Infinity (*p* = NS)

OD: odds ratio; DAS28: disease activity score using 28 joints; SDAI: simple disease activity index; MCP-1: monocyte chemoattractant protein-1; ESR: erythrocyte sedimentation rate; CRP: C-reactive protein; RF: rheumatoid factor; anti-CCP: anticyclic citrullinated peptide; NS: non-significant; TNF: tumor necrosis factor; IL-6: interleukin-6; JAKi: Janus kinase inhibitors; CTLA4-Ig: cytotoxic T lymphocyte-associated antigen-4 immunoglobulin fusion proteins; cDMARDs: conventional disease-modifying anti-rheumatic drugs; MTX: methotrexate. Normal ranges: CRP < 5 mg/L; ESR < 20 mm/h for women aged ≤50 years and <30 mm/h for women aged >50 years; ESR < 15 mm/h for men aged ≤50 years and < 20 mm/h for men aged >50 years; RF < 15 IU/mL; anti-CCP ≤ 20 CU; HAQ-DI < 1.44; BMI = 18.5–24. Sex, age >41/<40 years, age >65/<64 years, CRP, ESR, HAQ-DI, *n* = 182; age 41–64/<40 years, *n* = 144; RF, *n* = 124; anti-CCP, *n* = 122; medication (cDMARDs) numbers, *n* = 182; ultrasound grade type, *n* = 105; Anti-TNF, *n* = 165; Anti-IL-6, *n* = 165; JAKi, *n* = 165; CTLA4-Ig, *n* = 165; cDMARDs (MTX), *n* = 186; Comorbidities, *n* = 186. Medications include methotrexate, sulfasalazine, hydroxychloroquine, leflunomide, azathioprine, and cyclosporine. * *p* < 0.05; ** *p* < 0.001.

**Table 3:** Comparative analysis of physiological and biochemical, drug-related, and comorbidity factors in combined remission/low disease activity state based on different DAS28 scores.

	DAS28-ESR	DAS28-CRP	SDAI	DAS28-MCP-1
Female/Male	OR = 0.316 (*p* = 0.009 *)	OR = 0.909 (p = NS)	OR = 1.017 (*p* = NS)	OR = 1.062 (*p* = NS)
Age (≦40/>40)	OR = 0.206 (*p* = NS)	Infinity (*p* = NS)	OR = 0.714 (*p* = NS)	OR = 1.123 (*p* = NS)
Age (≦40/41–64)	OR = 0.180 (*p* = NS)	Infinity (*p* = NS)	OR = 0.689 (*p* = NS)	OR = 0.950 (*p* = NS)
Age (≦64/>64)	OR = 1.664 (*p* = NS)	Infinity (*p* = NS)	OR = 1.114 (*p* = NS)	OR = 2.009 (*p* = NS)
RF (Negative/positive)	OR = 1.181 (*p* = NS)	OR = 0.933 (*p* = NS)	OR = 0.993 (*p* = NS)	OR = 0.779 (*p* = NS)
Anti-CCP (Negative/positive)	OR = 0.476 (*p* = NS)	Infinity (*p* = NS)	OR = 0.384 (*p* = NS)	OR = 0.428 (*p* = 0.033 *)
CRP (Normal/high)	OR = 11.10 (*p* = 0.002 **)	Infinity (*p* = NS)	OR = 1.661 (*p* = NS)	OR = 0.995 (*p* = NS)
ESR (Normal/high)	OR = 14.417 (*p* < 0.001 ***)	OR = 0.661 (*p* = NS)	OR = 1.103 (*p* = NS)	OR = 1.613 (*p* = NS)
HAQ-DI (Normal/high)	OR = 5.189 (*p* = 0.018 *)	Infinity (*p* = NS)	OR = 8.000 (*p* = 0.001 **)	OR = 3.549 (*p* = 0.002 *)
Medication numbers (≦2/>2)	OR = 1.225 (*p* = NS)	OR = 3.281 (*p* = NS)	OR = 0.837 (*p* = NS)	OR = 1.662 (*p* = NS)
BMI (Normal/abnormal)	OR = 1.264 (*p* = NS)	OR = 0.380 (*p* = NS)	OR = 1.482 (*p* = NS)	OR = 1.324 (*p* = NS)
Ultrasound grade (≦1/>1)	OR = 1.372 (*p* = NS)	OR = 4.706 (*p* = NS)	OR = 1.506 (*p* = NS)	OR = 1.160 (*p* = NS)
Anti-TNF (User/non-user)	OR = 1.182 (*p* = NS)	OR = 1.540 (*p* = NS)	OR = 1.190 (*p* = NS)	OR = 1.378 (*p* = NS)
Anti-IL-6 (User/non-user)	OR = 1.513 (*p* = NS)	OR = 2.120 (*p* = NS)	OR = 0.926 (*p* = NS)	OR = 0.945 (*p* = NS)
Anti-JAK (User/non-user)	OR = 0.677(*p* = NS)	OR = 0.749 (*p* = NS)	OR = 1.006 (*p* = NS)	OR= 1.069 (*p* = NS)
CTLA4-Ig (Use/non-user)	OR = 0.747 (*p* = NS)	Infinity (*p* = NS)	OR = 0.783 (*p* = NS)	OR = 0.523 (*p* = NS)
cDMARDs (MTX/non-MTX)	OR = 1.356 (*p* = NS)	OR = 2.340 (*p* = NS)	OR = 1.814 (*p* = NS)	OR = 0.523 (*p* = NS)
Comorbidities (<2/≧2)	OR = 1.085 (*p* = NS)	Infinity (*p* = NS)	Infinity (*p* = 0.038)	Infinity (*p* < 0.001)

OD: odds ratio; DAS28: disease activity score by 28 joints; SDAI: simple disease activity index; MCP-1: monocyte chemoattractant protein-1; ESR: erythrocyte sedimentation rate; CRP: C-reactive protein; RF: rheumatoid factor; anti-CCP: anticyclic citrullinated peptide; NS: Non-significant; TNF: Tumor necrosis factor; IL-6: Interleukin-6; JAKi: Janus kinase inhibitors; CTLA4-Ig: Cytotoxic T lymphocyte-associated antigen-4 immunoglobulin fusion proteins; cDMARDs: conventional disease-modifying anti-rheumatic drugs; MTX: methotrexate. Normal ranges: CRP < 5 mg/L; ESR < 20 mm/h for women aged ≤50 years and < 30 mm/h for women aged >50 years; ESR < 15 mm/h for men aged ≤50 years and < 20 mm/h for men aged >50 years; RF < 15 IU/mL; anti-CCP ≤ 20 CU; HAQ-DI < 1.44; BMI = 18.5–24. Sex, age > 41/<40 years, age > 65/<64 years, CRP, ESR, HAQ-DI, *n* = 182; age 41–64/<40 years, *n* = 144; RF, *n* = 124; anti-CCP, *n* = 122; medication (cDMARDs) numbers, *n* = 182; ultrasound grade type, *n* = 105; Anti-TNF, *n* = 165; Anti-IL-6, *n* = 165; JAKi, *n* = 165; CTLA4-Ig, *n* = 165; cDMARDs (MTX), *n* = 186; Comorbidities, *n* = 186. Medications include methotrexate, sulfasalazine, hydroxychloroquine, leflunomide, azathioprine, and cyclosporine. * *p* < 0.05; ** *p* < 0.001; *** *p* < 0.0001.

**Table 4:** Correlation between total ultrasound scores and surface enzymes on B lymphocytes or monocytes (A) and comparison of surface enzymes on B lymphocytes or monocytes between different ultrasound grade types (B).

(A)	B ST3	B Neu3	M ST3	M Neu3	B Ratio	M Ratio
Spearman r	0.017	−0.01	−0.014	−0.02	−0.032	0.015
*p* value	NS	NS	NS	NS	NS	NS
**(B)**	**B ST3**	**B Neu3**	**M ST3**	**M Neu3**	**B Ratio**	**M Ratio**
*p* value	NS	NS	NS	NS	NS	NS

B: B cells; M: monocytes; ST3: α-2,3-sialyltransferase 1; Neu3: neuraminidase-3; B ratio: ST3/Neu3 ratio in B cells; M ratio: ST3/Neu3 ratio in monocytes.

**Table 5:** Correlation between total ultrasound scores and various DAS28 formulas.

	DAS28-ESR	DAS28-CRP	SDAI	DAS28-MCP-1
Pearson r	0.262	0.167	0.064	−0.043
*p* value	0.008 **	0.091	0.518	0.670

** *p* value < 0.01.

**Table 6:** Correlation between DAS28/SDAI scores and surface enzymes on monocytes.

	M ST3	M Neu3	M ST3/Neu3 Ratios
DAS28-ESR > 5.1	rho = 0.609,		rho = 0.662,
	*p* = 0.012		*p* = 0.005
3.3 < SDAI ≦ 11			rho = 0.342,
			*p* = 0.048
DAS28-MCP-1 > 4.8		rho = 0.727,	
		*p* = 0.011	

All correlations were analyzed using Spearman’s analysis. M: monocytes; ST3: α-2,3-sialyltransferase 1; Neu3: neuraminidase-3; M ST3/Neu3 ratio: monocyte α-2,3-sialyltransferase 1/neuraminidase-3 ratio.

**Table 7:** Clinical, laboratory, and ultrasound data at various stages of the disease.

	M0 (*n* = 80)	M6 (*n* = 52)	M12 (*n* = 50)
CRP (mg/L)	4.50 ± 10.36 (0.2–88.86)	3.25 ± 4.05 (0.2–15.43)	4.76 ± 6.87 (0.2–31.46)
ESR (mm/h)	25.70 ± 24.98 (1–140)	21.75 ± 21.98 (1–140)	22.45 ± 22.73 (1–140)
TJC	4.10 ± 3.53 (0–17)	4.19 ± 3.57 (0–17)	4.07 ± 3.70 (0–23)
SJC	3.45 ± 2.79 (0–11)	2.94 ± 2.86 (0–13)	3.13 ± 2.06 (0–8)
DAS28-ESR	3.97 ± 0.99 (0.42–6.62)	3.76 ± 0.92 (1.64–5.91)	3.83 ± 0.89 (1.25–5.83)
DAS28-CRP	4.15 ± 0.91 (1.40–6.60)	4.07 ± 0.88 (1.38–5.66)	4.17 ± 0.85 (1.38–5.94)
SDAI	14.80 ± 6.99 (0.02–35.20)	14.44 ± 6.74 (0.02–30.23)	14.44 ± 7.24 (0.02–45.02)
DAS28-MCP-1	3.68 ± 0.84 (1.70–6.46)	3.71 ± 0.80 (1.39–5.45)	3.48 ± 0.95 (0.66–5.15)
HAQ-DI	0.65 ± 0.89 (0–4)	0.59 ± 0.82 (0–4)	0.61 ± 0.80 (0–2.67)
RF (IU/mL)	162.3 ± 269.7 (5–1200)		132.7 ± 226.8 (9.1–1200)
Anti-CCP (CU)	705.8 ± 1065.9 (4.6–6017.8)		585.7 ± 896.8 (4.6–2776.7)
Total ultrasound scores	5.67 ± 4.89 (0–22)		4.96 ± 4.42 (0–20, *n* = 48 *)
B ST3	22.85 ± 23.39 (3.35–167)	26.65 ± 24.54 (6.35–162)	22.91 ± 25.87 (8.73–162)
B Neu3	24.28 ± 29.06 (3.41–207)	26.71 ± 27.93 (6.38–192)	26.23 ± 38.25 (8.21–220)
M ST3	187.98 ± 1188.22 (12.2–9910)	118.84 ± 595.59 (9.68–4024)	29.04 ± 19.73 (7.14–97)
M Neu3	40.32 ± 38.75 (0–258)	47.18 ± 83.00 (11.4–508)	29.89 ± 27.44 (7.72–178)
B ST3/Neu3 ratio	1.00 ± 0.17 (0.18–1.53)	1.03 ± 0.15 (0.81–1.66)	0.98 ± 0.16 (0.50–1.38)
M ST3/Neu3 ratio	1.12 ± 0.46 (0.57–3.78)	1.12 ± 1.06 (0.14–7.92)	1.06 ± 0.31 (0.45–2.17)

B: B cell; M: monocytes; ST3: α-2,3-sialyltransferase 1; Neu3: neuraminidase-3; M ST3/Neu3 ratio: monocyte α-2,3-sialyltransferase 1/neuraminidase-3 ratio. * The number of patients in the ultrasound score is only 48 at month 12.

## Data Availability

The original contributions presented in the study are included in the article, further inquiries can be directed to the corresponding author.
